# Development of a model for anemia of inflammation that is relevant to critical care

**DOI:** 10.1186/s40635-019-0261-2

**Published:** 2019-07-25

**Authors:** Margit Boshuizen, Robin van Bruggen, Sebastian A. Zaat, Marcus J. Schultz, Eli Aguilera, Ana Motos, Tarek Senussi, Francesco Antonio Idone, Paolo Pelosi, Antonio Torres, Gianluigi Li Bassi, Nicole P. Juffermans

**Affiliations:** 1 Department of Intensive Care Medicine, Amsterdam UMC, University of Amsterdam, Meibergdreef 9, Amsterdam, 1105 AZ the Netherlands; 2 Department of Blood Cell Research, Sanquin Research and Landsteiner Laboratory, Amsterdam UMC, University of Amsterdam, Amsterdam, 1066 CX The Netherlands; 3 Department of Microbiology, Center for Infection and Immunity Amsterdam (CINIMA), Academic Medical Center, University of Amsterdam, Amsterdam, 1105 AZ The Netherlands; 4 Department of Pulmonary and Critical Care Medicine, Thorax Institute, Hospital Clínic, 08036 Barcelona, Spain; 5 Department of Surgical Sciences and Integrated Diagnostics (DISC), San Martino Policlinico Hospital - IRCCS for Oncology, 16132 Genova, Italy

**Keywords:** Animal model, Anemia of inflammation, Infection, ICU, Iron

## Abstract

**Background:**

Anemia of inflammation (AI) is common in critically ill patients. Although this syndrome negatively impacts the outcome of critical illness, understanding of its pathophysiology is limited. Also, new therapies that increase iron availability for erythropoiesis during AI are upcoming. A model of AI induced by bacterial infections that are relevant for the critically ill is currently not available. This paper describes the development of an animal model for AI that is relevant for critical care research.

**Results:**

In experiments with rats, the rats were inoculated either repeatedly or with a slow release of *Streptococcus pneumoniae* or *Pseudomonas aeruginosa*. Rats became ill, but their hemoglobin levels remained stable. The use of a higher dose of bacteria resulted in a lethal model. Then, we turned to a model with longer disease duration, using pigs that were supported by mechanical ventilation after inoculation with *P. aeruginosa.* The pigs became septic 12 to 24 h after inoculation, with a statistically significant decrease in mean arterial pressure and base excess, while heart rate tended to increase. Pigs needed resuscitation and vasopressor therapy to maintain a mean arterial pressure > 60 mmHg. After 72 h, the pigs developed anemia (baseline 9.9 g/dl vs. 72 h, 7.6 g/dl, *p* = 0.01), characterized by statistically significant decreased iron levels, decreased transferrin saturation, and increased ferritin. Hepcidin levels tended to increase and transferrin levels tended to decrease.

**Conclusions:**

Using pathogens commonly involved in pulmonary sepsis, AI could not be induced in rats. Conversely, in pigs, *P. aeruginosa* induced pulmonary sepsis with concomitant AI. This AI model can be applied to study the pathophysiology of AI in the critically ill and to investigate the effectivity and toxicity of new therapies that aim to increase iron availability.

**Electronic supplementary material:**

The online version of this article (10.1186/s40635-019-0261-2) contains supplementary material, which is available to authorized users.

## Background

Anemia is a hallmark of critical illness, and inflammation is thought to contribute to the development of anemia in the majority of critically ill patients. Thereby, anemia of inflammation (AI) is common in the intensive care unit (ICU) [1]. The pathophysiology of AI is multifactorial and includes a shortened red blood cell (RBC) life span caused by erythrophagocytosis [2], as well as a decreased erythropoiesis [3]. Erythropoiesis is impaired due to inflammatory cytokines that decrease erythroid precursor proliferation and erythropoietin levels [4]. Also, serum iron levels are low in AI due to increased levels of hepcidin, which is the iron-regulating hormone that is produced in response to inflammatory cytokines [5]. Hepcidin causes degradation of the iron exporter ferroportin, resulting in sequestration of iron inside cells and subsequent low plasma iron [6]. Thereby, in contrast to iron deficiency anemia, AI patients do not have a lack of iron, but rather a decreased iron availability.

Regardless of the cause, anemia in the critically ill is associated with adverse outcome [7] and AI occurs early after ICU admission [8]. AI is treated with RBC transfusions. In sepsis, half of the patients require RBC transfusion within the first 24 h of ICU admission [8]. However, RBC transfusion is associated with morbidity and mortality in the critically ill [9], calling for alternative therapies. In the last decade, new therapies to treat AI by targeting the iron metabolism are in development, such as hepcidin inactivators [10–12], hepcidin production inhibitors [13, 14], interleukin 6 (IL-6) inhibitor [15], and IL-6 receptor blockers [16–18]. These therapies aim to increase the amount of iron available for erythropoiesis, which could potentially reduce the amount of transfusions and improve the outcome of critical illness. However, such interventions may also have drawbacks, such as slow resolution of infection or acquisition of new infections, as bacteria use iron for their growth [19]. Therefore, new therapies for critically ill patients that increase iron availability should preferably be tested in models of AI that are caused by bacterial infections that are relevant for the critically ill. The currently available AI animal models are mainly non-infectious models, including heat-killed *Brucella abortus* [2], zymosan [20], cytokines [11, 21], or peptidoglycan-polysaccharide [22]. Infectious AI animal models show high variation due to technical difficulties [23, 24], have only mild anemia [25, 26], or use parasitic infections that are not relevant for the critically ill [27–30]. Taken together, animal models for AI that mimic critical care illness are currently limited. Therefore, through a multi-national European collaboration, we aimed at developing an animal model of AI caused by pulmonary sepsis, evaluating the advantages and disadvantages of models in rats and pigs.

## Methods

### Rat experiments

Studies were approved by the Institutional Animal Care and Use Committee of the Amsterdam University Medical Centers, located at the Academic Medical Center, Amsterdam, Netherlands. All animal procedures were performed in compliance with Institutional Standards for Human Care and Use of Animal Laboratory Animals.

#### Experimental protocol

For all experiments, the bacterial inoculum was prepared as follows: an overnight bacterial culture was diluted in fresh medium. The bacteria were cultured to logarithmic growth phase at 37 °C. Then, the culture was centrifuged and the pellet was washed and resuspended in sterile saline. This bacterial suspension was diluted to the desired inoculum concentration, based on the optical density at 600 nm. The inoculum concentration was verified by the culture of 10-fold serial dilutions of the inoculum on agar plates.

**Fig. 1:**
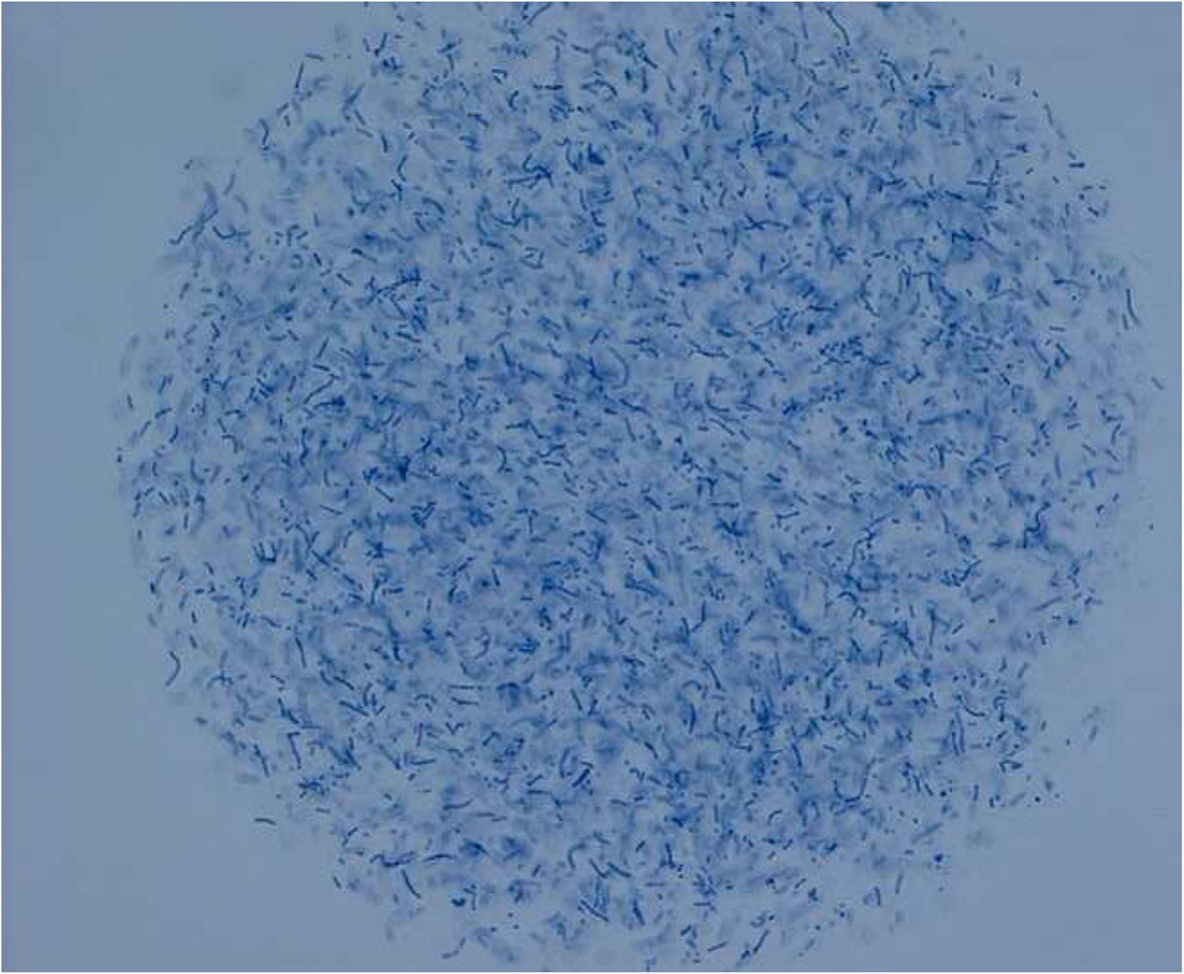
*Streptococcus pneumoniae* embedded in an agar bead. Richardson staining, × 400 magnification. Agar beads with *Streptococcus pneumonia* have a diameter of 50–200 μm. The size of these *S. pneumonia* beads is similar to the *Pseudomonas aeruginosa* agar beads in the original protocol [31].

Male Sprague-Dawley rats (Envigo, The Netherlands) were anesthetized with 3% isoflurane. After baseline blood sampling via a catheter in the tail vein, rats were intratracheally inoculated with a high dose of 10^8^ colony-forming units (CFU) of a log-phase culture of *Streptococcus pneumoniae* serotype 3 (ATCC 6303; Rockville, MD, USA) in a volume of 150 μL using a miniature nebulizer. A second group of rats was inoculated repeatedly, at day 0 and day 4, with 10^7^ CFU of a log-phase culture of *S. pneumoniae* in a volume of 150 μL. A third group of rats was inoculated with 10^6^ CFU of a log-phase culture of *S. pneumoniae* embedded in agar beads in a volume of 150 μL, from which bacteria are slowly released [31] (Fig. 1). These *S. pneumoniae* agar beads were prepared as described in the Additional file 1. Finally, additional rats were inoculated with high dose *Pseudomonas aeruginosa* (PA103, 10^9^ CFU; kindly provided by Iglewski Laboratory, Rochester, NY, USA) in a volume of 150 μL either in a bolus solution or via slow release, embedded in agar beads.

All rats were weighed daily. Supplemental fluid bolus (10 ml/kg Ringers Lactate) was given every 24 h intraperitoneally in case of severe illness, as indicated by > 10% weight loss compared to the previous day. Blood samples of 500 μl were taken via the tail vein at baseline and at day 3, 7, and 10 to measure the hemoglobin (Hb) level. No control group was used in these experiments. Hb levels were compared with the baseline measurement. After 7 or 14 days, rats were anesthetized by intraperitoneal injection of 90 mg/kg ketamine (Dechra, The Netherlands), 0.25 mg/kg dexmedetomidine (Orion Pharrma, Finland), and 0.5 mg/kg atropine (Centrafarm, The Netherlands) to bleed them via the inferior caval vein. The blood was collected into EDTA anti-coagulated tubes

#### Sample analysis

Hb levels were measured using the ScilVet abc + (scil animal care company GmbH, Germany). Of each rat, the two middles lobes of the right lung were homogenized in 1 ml sterile PBS. Serial 10-fold dilutions were plated on blood agar plates and incubated at 37 °C with 5% CO_2_. The number of CFUs was counted the next day.

### Pig experiment

This study was approved by the Institutional Review Board and Animal Ethics Committee of the University of Barcelona, Barcelona, Spain. The animal procedures were performed according to local Spanish guidelines for the use and care of animals.

#### Experimental protocol

A porcine ventilator-associated pulmonary sepsis model was used, as has been described before [32]. Five female large-white Landrace pigs of 31 ± 1.3 kg (range 29–32 kg) were used. Pigs were premedicated with intramuscular 2 mg/kg azaperone. Then, animals were induced with 2–2.5 mg/kg of propofol, orotracheally intubated with a 7.5-mm I.D. endotracheal tube and mechanically ventilated with a SERVO-i (Maquet, Wayne, NJ, USA). Pigs were ventilated in volume-control setting, with a tidal volume of 10 ml/kg, inspiratory fraction of oxygen 40%, and without positive end-expiratory pressure. Respiratory rate was adjusted every 6 h to maintain normocapnia (40–45 mmHg PaCO_2_). An arterial line was inserted into the femoral artery to monitor systemic arterial pressure and to collect blood samples. A central venous catheter was surgically placed in the jugular vein for intravenously fluid and drug administration. A no. 8 Foley catheter was placed into the bladder through surgical mini-pelvectomy. Ceftriaxon (1 g) was administrated intravenously 30 min before intubation and 50 mg/kg was given every 12 h to prevent pneumonia caused by oropharyngeal flora. Following surgical preparation and stabilization—approximately 4 h after intubation—the pigs were bronchoscopically inoculated with 15 mL of 10^8^ CFU of a log-phase culture of *Pseudomonas aeruginosa* (subtype ATCC 27853, ceftriaxone resistant) into each lobe. The inoculum was prepared as described above. Ringer lactate and 0.9% NaCl solution were infused to maintain fluid balance. The animals received 2 l of 0.9% NaCl daily. Also, boluses of 20 ml/kg were given when the pig was thought to be fluid responsive. The fluid therapy targeted urine output ≥ 0.5 m/kg/h. Blood was drawn via the arterial line into EDTA anti-coagulated and serum separating tubes (BD™ Vacutainer™ SST™ II Advance Tubes). Samples were centrifuged for 10 min, at 1750×*g* and the serum was stored at − 80 °C. Blood samples were taken at baseline (just prior to inoculation) and every 12 h thereafter, until 72 h. Bronchoalveolar lavage (BAL) was performed bronchoscopically with 40 ml saline in the right medium lobe at baseline, 24 and 72 h thereafter. The animals were euthanized 72 h after intubation using intravenous overdosing propofol, potassium.

#### Blood sampling and analysis

Full blood count and hemoglobin was assessed every 24 h through a hematocytometer (Siemens Advia 2021i, Erlangen, Germany). At the same time points, serum iron was measured using a colorimetric assay (Sekisui Diagnostics, Lexington, MA). Total iron-binding capacity (TIBC) was measured using a colorimetric assay (Pointe Scientific). Transferrin levels were calculated using the formula TIBC (μmol/L)/25.1. Transferrin saturation was calculated with the formula serum iron/(25.1 × total transferrin) × 100%. Ferritin (LsBio), hepcidin (LsBio), and interleukin 6 (IL-6) levels (Millipore Iberica, S.A., Madrid, Spain) were measured by enzyme-linked immunosorbent assay kits. Lung tissue was homogenized and cultured on agar plates at 37 °C with 5% CO_2_. The number of CFUs was counted the next day.

#### Statistical analysis

The data is expressed as median ± IQR. Differences between time points were analyzed using a paired samples *t* test or a Wilcoxon signed-ranks test, for normally and not normally distributed parameters, respectively. Statistical significance was considered to be at *p* ≤ 0.05. All statistical analyses were performed using IBM SPSS Statistics 24.

## Results

### Rat experiments

#### Streptococcus pneumoniae pneumonia in rats does not cause AI

**Fig. 2:**
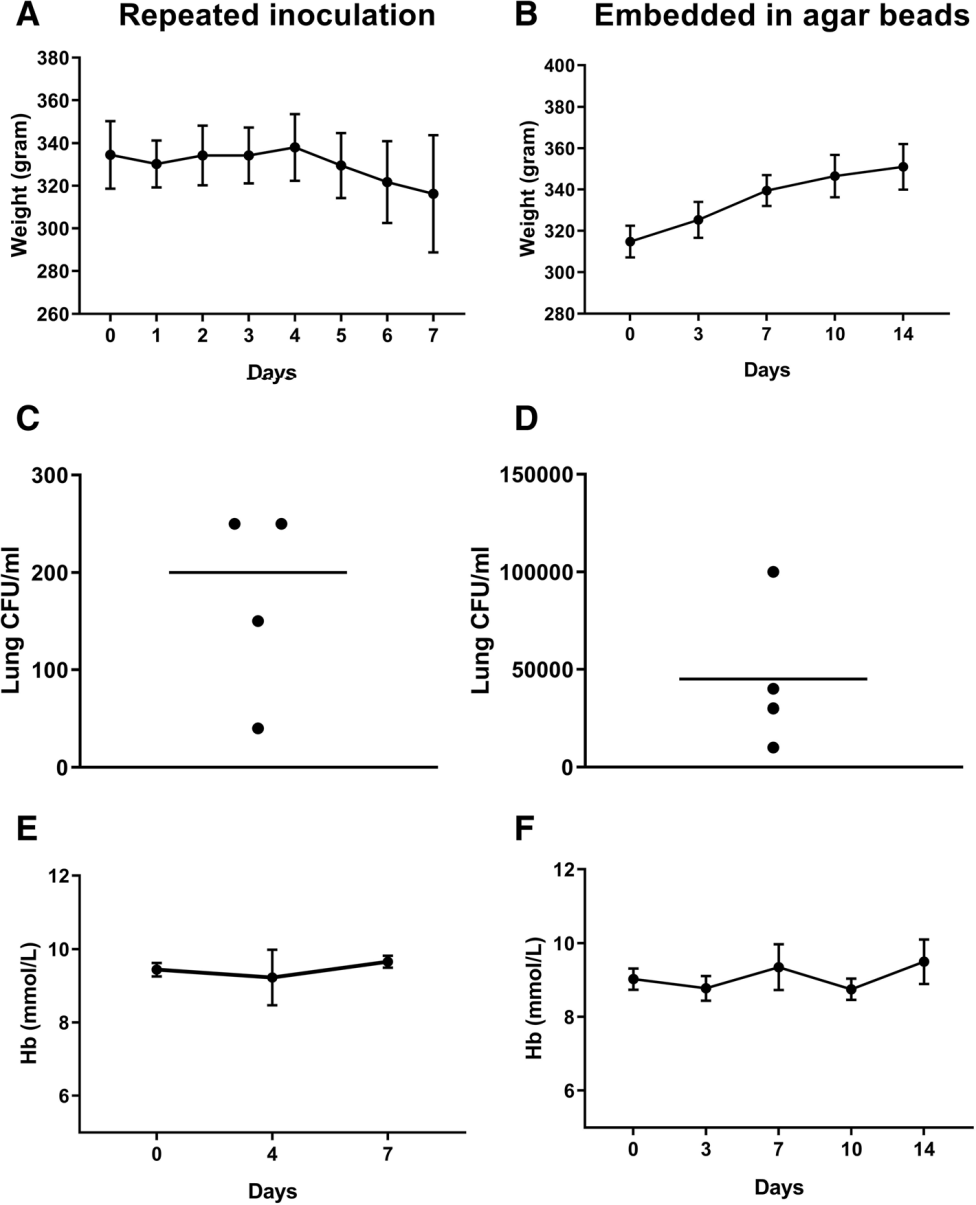
Failed attempts to establish rat AI model using *S. pneumoniae*. Daily weight (**a**), lung CFU/ml after 7 days (**b**), and Hb levels (**c**) of rats inoculated with a log-phase culture of 10^7^ CFU *S. pneumoniae* at day 0 and day 4. The daily weight (**d**), lung CFU/ml (**e**), and Hb levels (**f**) of rats inoculated with a log-phase culture of 10^6^ CFU *S. pneumoniae* embedded in agar beads. *N* = 4 and data are expressed as mean + SD (*n* = 4). ***P < 0.001, **P < 0.01, *P < 0.05

Rats infected with a bolus dose of 10^8^ CFU *S. Pneumoniae* appeared very ill, with 10% weight loss and a ruffled fur at 24 h after inoculation. The animals died the second day, prior to the development of anemia. Rats inoculated with 10^7^ CFU *S. Pneumoniae* did not show a decrease in weight. At day 4, these rats were inoculated again with 10^7^ CFU *S. Pneumoniae*, and from then on, the animals started to lose weight (Fig. 2a). At day 7, the rats appeared ill, with lethargic behavior and ruffled fur. The animals showed macroscopic pneumonia, and there were still bacteria present in the lungs, indicating that the infection was not cleared (Fig. 2b). However, Hb levels were not decreased at 7 days compared to baseline (Fig. 2c). Next, an approach was taken using bacteria embedded in beads, with the aim to induce a slow releasing reservoir resulting in prolonged unresolved infection. Rats inoculated with 10^6^ CFU *S. pneumoniae* embedded in agar beads did not lose weight during the experiment, but even seemed to gain weight (Fig. 2d). At 14 days, macroscopic lesions were visible in the lungs and there were still bacteria present in the lungs (Fig. 2e). However, Hb levels did not decrease during the 14 days experiment (Fig. 2c). In addition, rats inoculated with 10^9^ CFU *P. aeruginosa* or 10^9^ CFU *P. aeruginosa* embedded in agar beads did not show any decrease in Hb level either (data not shown).

### Pig experiments

#### *Pseudomonas aeruginosa* causes pneumonia and shock in pigs

**Fig. 3:**
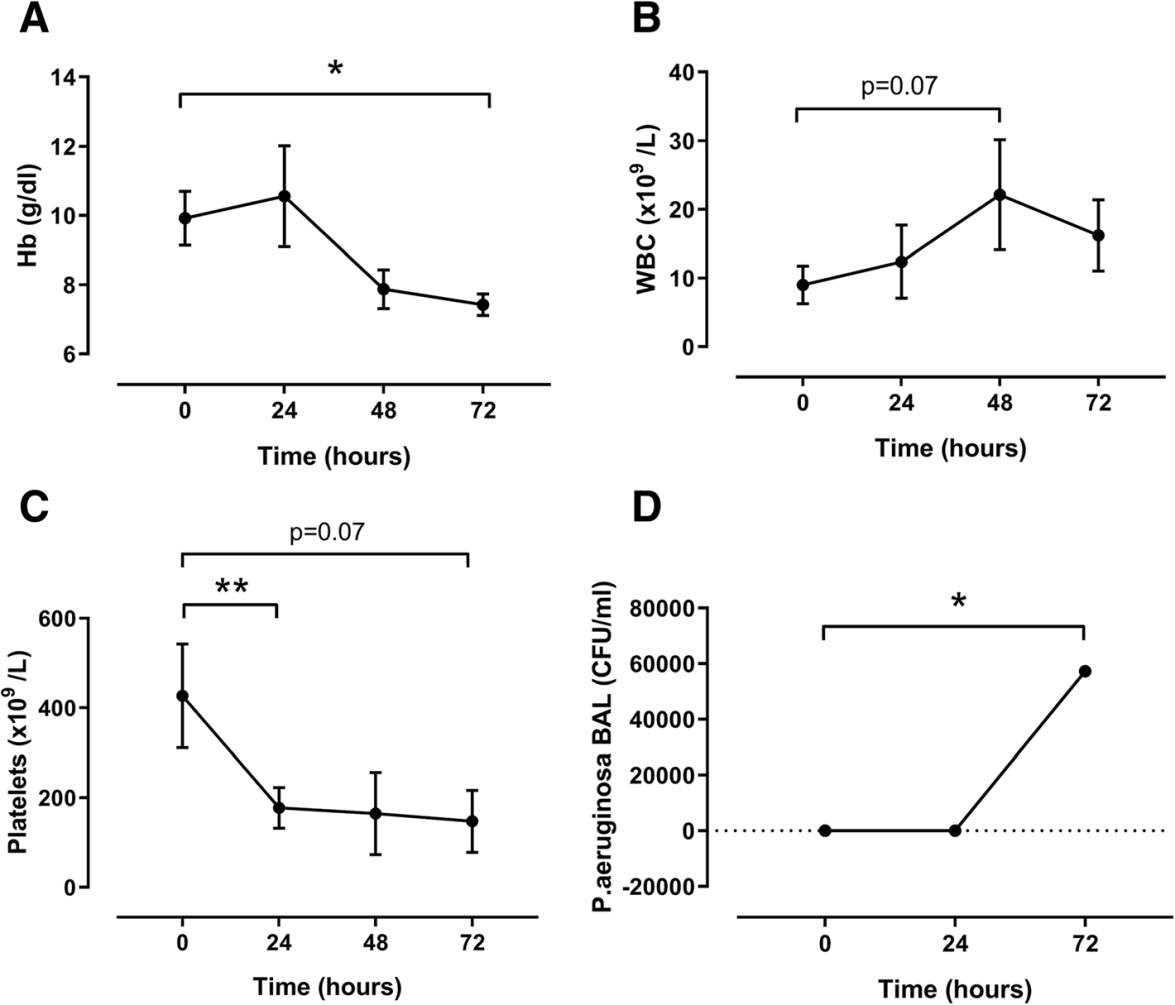
Characteristics of a pig pneumosepsis model. Hb levels (**a**), WBC counts (**b**), platelet counts (**c**), and *P. aeruginosa* concentration in the BAL fluid (**d**) in pigs inoculated with 15 mL of 10^8^ CFU of a log-phase culture of *P. aeruginosa* into each lung lobe. *N* = 5 and data are expressed as mean + SD (*n* = 5). ***P < 0.001, **P < 0.01, *P < 0.05

Between 12 and 24 h after inoculation, pigs presented signs of severe respiratory infection. Table 1 shows the hemodynamic parameters of the pigs at several time points during the experiment. The mean arterial pressure decreased over time, from 80 mmHg (76–85) at baseline to 67 mmHg (63–71) at 24 h (*p* = 0.02), while the heart rate (*p* = 0.07) and noradrenaline infusion rate (*p* = 0.07) tended to increase in the first 24 h. Arterial base excess decreased between baseline and 12 h, from 7.6 mmol/L (5.7–9.7) to 2.8 mmol/L (− 1.1–6.4) (*p* = 0.03) (Table 1). Also, platelets decreased in the first 24 h from 338 × 10^9^/L (330–568) to 205 × 10^9^/L (123–219) (*p* = 0.004) and remained low throughout the experiment. White blood cell (WBC) counts tended to increase over time, from 8.8 × 10^9^/L (6.5–11.6) at baseline to 23.8 × 10^9^/L (12.8–29.9) at 48 h (*p* = 0.07). CFU counts increased during the experiment, from 0 at baseline until 4.6 log CFU/ml (3.7–5.9) (*p* = 0.04) in BAL fluid at 72 h (Fig. 3).

**Table 1:** Hemodynamic parameters of pigs with pneumosepsis

Time (h)	MAP (mmHg)	HR (beats/min)	Noradrenalin (μg/kg/min)	Arterial BE (mmol/L)
0	80 (4)	65 (29)	0 (0)	7.6 (2.1)
12	81 (12)	130 (41)	0.46 (0.46)	2.8 (4.6)
24	67 (5)	97 (28)	3.7 (3.1)	4.4 (5)
48	74 (12)	79 (13)	8.7 (14.8)	7.6 (2.7)
72	83 (13)	55 (11)	1.6 (2.1)	7.4 (2.2)
*P* value	0.02*	0.07^#^	0.07*	0.03^#^

*BE* base excess, *MAP* mean arterial pressure, *HR* heart rate. *N* = 5 and data are expressed as mean (SD)

^#^*P* values are calculated for the difference between baseline and 12 h

*P values are calculated for the difference between baseline and 24 h

#### *Pseudomonas aeruginosa* pneumonia causes AI in pigs

**Fig. 4:**
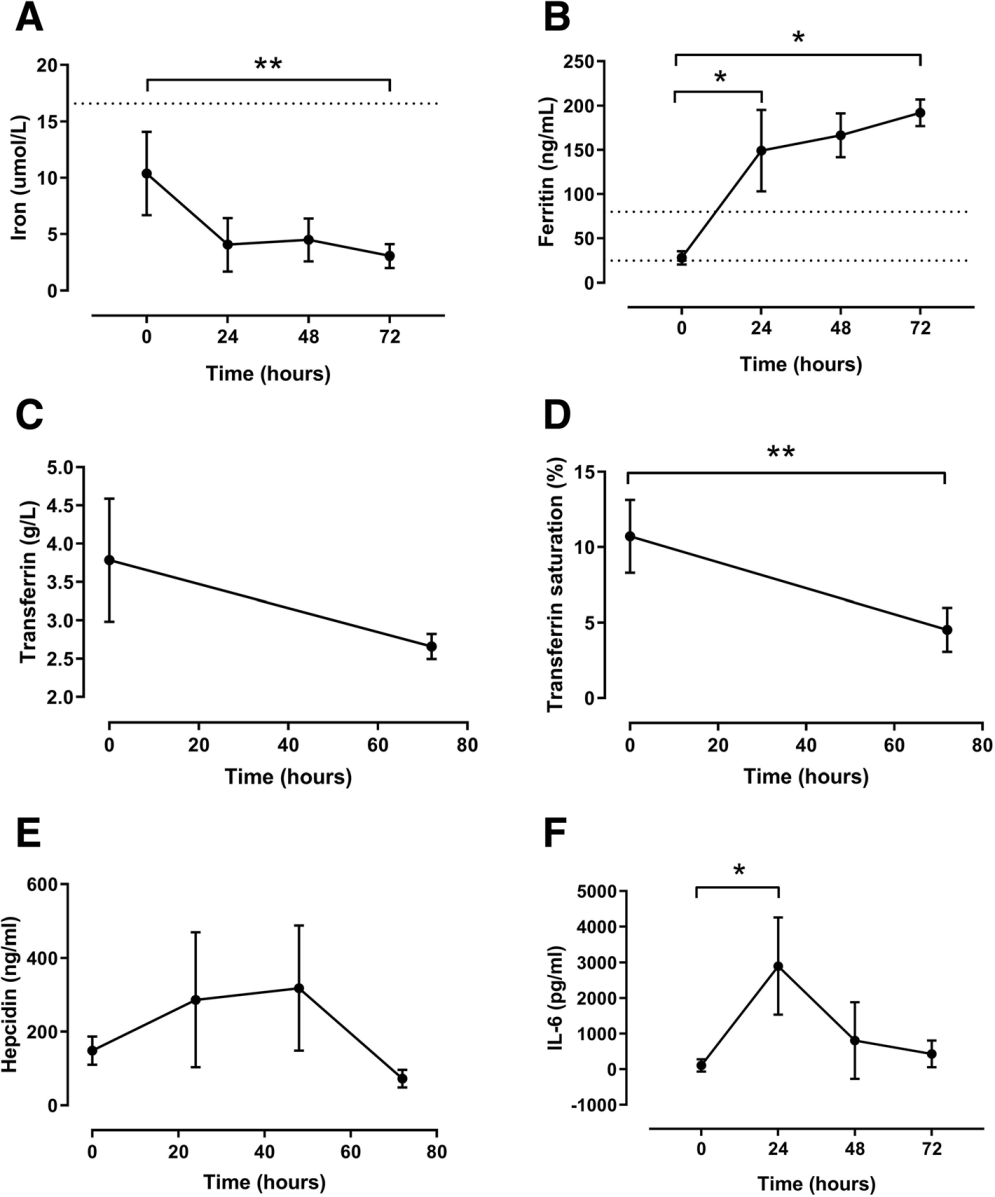
Iron parameters in pigs with pneumosepsis. Levels of iron (**a**), ferritin (**b**), transferrin (**c**), transferrin saturation (**d**), hepcidin (**e**), and IL-6 (**f**) in pigs inoculated with 15 mL of 10^8^ CFU of a log-phase culture of *P. aeruginosa* into each lung lobe. *N* = 5 and data are expressed as mean + SD (*n* = 5). ***P < 0.001, **P < 0.01, *P < 0.05. Dotted lines represent reference levels

Hb levels at baseline were 9.9 g/dl (9.2–10.7) and decreased significantly over time to 7.6 g/dl (7.1–7.7) after 72 h (*p* = 0.01) (Fig. 3). Serum iron level at baseline was already lower than normal for pigs (9.5 μmol/L (6.8–14.4)) and decreased significantly throughout the experiment to 3.5 μmol/L (1.9–4.1) after 72 h (*p* = 0.01). Also, transferrin saturation decreased significantly over time from 10.4% (8.6–13.0) at baseline to 4.7% (2.9–6.0) after 72 h (*p* < 0.01). Transferrin levels tended to decrease during the experiment from 3.6 g/L (3.0–4.7) at baseline to 2.6 g/L (2.5–2.8) at *T* = 72 h (*p* = 0.08). Ferritin levels increased significantly from 25.2 ng/ml (21.1–36.4) at baseline to 188.6 ng/ml (178.0–207.8) after 72 h (*p* = 0.04). Hepcidin levels slightly increased during the experiment from 151.2 ng/ml (110.1–185.5) at baseline to 359.9 ng/ml (138.8–476.2) after 48 h (*p* = 0.08). Finally, IL-6 increased significantly from 31 pg/ml (17–241) to 2698 pg/ml (1713–4180) at *T* = 24 h (*p* = 0.04) (Fig. 4).

## Discussion

Initially, the use of rodents was evaluated for the development of this AI model, because of the lower costs and because of ample experience with these animals. However, efforts to develop a rat model for AI due to *S. pneumoniae* or *P. aeruginosa* pneumonia were unsuccessful. The rats did not survive inoculation of high dose bacteria, whereas a lower, repeated dose of bacteria or slow-release infection with bacteria embedded in agar beads did not result in AI, despite the presence of unresolved infection at day 14. This suggests that it is not feasible to induce AI due to prolonged severe infection in rats with the use of pathogens commonly encountered in the critically ill. Consequently, we switched to an existing pig model. This model is a model of longer lasting pneumosepsis induced by *P. aeruginosa* and has been detailed elsewhere [32]. However, the anemia status in this model has never been described.

We found that prior to illness induction, the pigs have low iron and ferritin levels, compatible with iron deficiency at baseline. However, the pigs developed anemia over time during the sepsis. The decrease in Hb was due to AI, exemplified by hypoferremia, decreased transferrin saturation, and increased ferritin levels, associated with an increased IL-6 and hepcidin levels. Also, serum transferrin levels tended to decrease due to the inflammation. Taken together, these pigs show all the characteristics of AI [33]. The main strength of this model is that it is very similar to the ICU setting since the pigs had severe pneumosepsis, requiring mechanical ventilation and hemodynamic support. The animals developed kidney injury and acute respiratory distress syndrome showing that this is a model for septic shock with multi-organ failure [32, 34, 35]. Other strengths of this model are the comparability to AI in humans and the possibility of a long-term study. Future animal studies could use this pig AI model to pre-test the efficacy and toxicity before the start of trials with new agents for AI in the critically ill. Also, as the cardiac output of pigs almost equals that of humans [36], dose-finding medication studies can be done in this model as well. However, as the pigs that we used had a mild iron-deficiency at baseline, we would recommend to correct this iron deficiency by an intramuscular iron injection for studies to AI [37]. Further, compared to patients on the ICU that receive mechanical ventilation for weeks, this model is still a short-term model.

## Conclusions

This study describes an AI model relevant for critical care. This model can be used to study the pathophysiology of AI during critically illness as well as the effect of new therapies for AI that aim to increase iron availability. Using this model, both the effect on anemia as well as the effect on immune host response, bacterial resolution, and organ failure can be studied.

## Additional file

Additional file 1: Protocol for embedding Streptococcus pneumonia in agar beads. (DOCX 14 kb)

## Data Availability

The datasets used and/or analyzed during the current study are available from the corresponding author on reasonable request.

## Abbreviations

AI: Anemia of inflammation
BAL: Bronchoalveolar lavage
BE: Base excess
CFU: Colony-forming unit
Hb: Hemoglobin
HR: Heart rate
ICU: Intensive care unit
IL-6: Interleukin 6
MAP: Mean arterial pressure
RBC: Red blood cell
TIBC: Total iron-binding capacity
