# Supplementary material for: Development of a model for anemia of inflammation that is relevant to critical care
Source: Intensive Care Med Exp. 2019 Jul 25;7(Suppl 1):47. doi: 10.1186/s40635-019-0261-2 (PMC6658638; doi:10.1186/s40635-019-0261-2)
Supplement: Supplementary file 1 — Protocol for embedding Streptococcus pneumonia in agar beads. (DOCX 14 kb) [file 40635_2019_261_MOESM1_ESM.docx]

**Supplement**

*Streptococcus pneumoniae* agar beads were prepared as described before [31], with some adaptations to the protocol to be able to embed the *S. pneumonia* alive in the agar beads.

Protocol for embedding *Streptococcus pneumonia* in agar beads:

-Take a scratch with a loop from a frozen stock of *Streptococcus pneumoniae* serotype 3 (ATCC 6303; Rockville, MD, USA) and put into 6 ml brain heart infusion (BHI) medium in a 15 ml tube. Incubate stationary overnight (approximately for 16 hours) in the incubator (37°C).

-Measure the optical density (OD) at 600nm and transfer 2 OD equivalents (approximately 2 ml) of the overnight culture into 3 separate tubes with 18 ml fresh BHI medium of 37^o^C.

-Microwave the autoclaved BHI medium with 1.5% agar until the agar has been dissolved and cool the agar in a water bath until 42°C. Also preheat 200 mL sterile heavy mineral oil (Sigma-Aldrich) in an Erlenmeyer flask in the water bath until 42°C.

-Incubate the bacterial suspensions at 37°C until log phase is reached (about 1 hour).

-Collect the bacteria by centrifugation at 2700 x *g* for 10 min at room temperature and brake with minimal deceleration. Discard the supernatant and resuspend the bacterial pellets of the tubes in 6 ml of sterile PBS.

-Mix 6 ml of the bacterial suspension with 12 ml BHI medium containing 1.5% agar of 42°C.

-Add the 18 ml mix to the 200 ml heavy mineral oil of 42°C – while stirring (there must be an vortex visible in the oil), and keep stirring for 6 min at room temperature to form the beads.

-Cool the mix to 4°C, while stirring at minimum speed, for 35 minutes.

-Let the agar beads in oil rest on ice for 20 minutes.

-Transfer the agar beads-oil mixture into 50 mL Falcon tubes and centrifuge at 2700 x *g*, for 15 minutes at 4°C.

-Discard the mineral oil and add sterile PBS, centrifuge at 2700 x *g* , for 15 minutes at 4°C. Repeat this wash cycle 5 more times. After the final wash cycle, resuspend the agar beads in 5 ml sterile PBS.

-Take 100 µL of the agar beads and make 10-fold serial dilutions to 10^-6^.

-Spread the dilutions on agar plates with a sterile glass spreader – this destroys the beads and spreads the bacteria – and incubate the plates at 37°C.

-Store the agar beads overnight at 4°C.

-Count the number of CFUs on the agar plates the next morning and determine the *S. pneumonia* concentration in the beads (CFU/ml). Dilute the agar bead suspension to the desired concentration and inoculate the rat with 200 µL.

NB. the size of the agar beads is 50-200 µm (figure 1).
